# *Herba Epimedii*: Anti-Oxidative Properties and Its Medical Implications

**DOI:** 10.3390/molecules15117861

**Published:** 2010-11-03

**Authors:** Stephen Cho Wing Sze, Yao Tong, Tzi Bun Ng, Chris Lok Yin Cheng, Ho Pan Cheung

**Affiliations:** 1 School of Chinese Medicine, LKS Faculty of Medicine, The University of Hong Kong, 10 Sassoon Road, Pokfulam, Hong Kong SAR, China; E-Mail: stephens@hku.hk (S.C.W.S.); 2 School of Biomedical Sciences, Faculty of Medicine, The Chinese University of Hong Kong, Shatin, Hong Kong SAR, China; E-Mail: tzibunng@cuhk.edu.hk (T.B.N.)

**Keywords:** *Herba Epimedii*, icarrin, anti-oxidant, flavonoids, polysaccharides, vitamin C

## Abstract

*Herba Epimedii* is a Chinese herbal medicine with proven efficacy in treating cardiovascular diseases and osteoporosis, and in improving sexual and neurological functions. This efficacy is found to be related to the potent anti-oxidative ability of *Herba Epimedii* and its flavonoid components, with icarrin as the main effective constituent, along with polysaccharides and vitamin C. These ingredients have been proven to be effective against oxidative-stress related pathologies (cardiovascular diseases, Alzheimer’s disease and inflammation) in animal rodent models and *in vitro* studies. Their anti-oxidative properties are found to be related to an inductive effect on endogenous free-radical scavenging enzymes such as catalase and glutathione peroxidase and the inherent electron-donating ability of flavonoids.

## 1. Introduction

**Figure 1:**
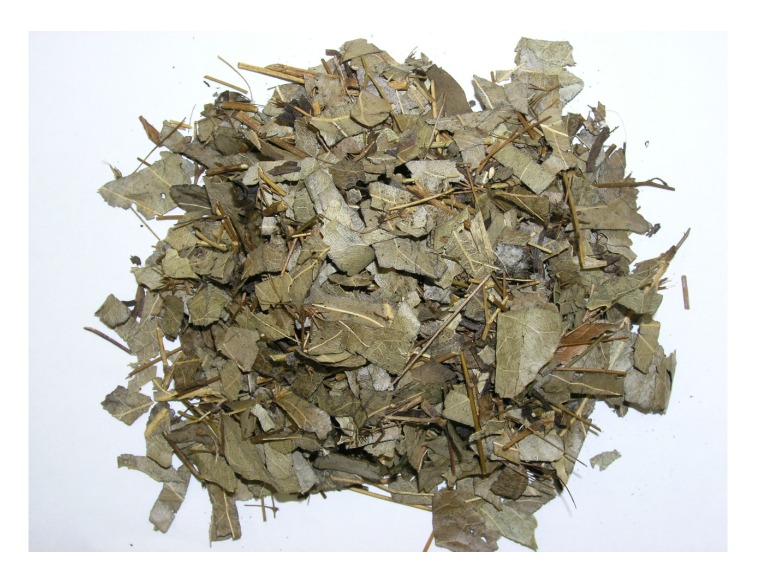
Photo of *Herba Epimedii*

Chinese herbal medicine has been widely used for centuries for the treatment of different diseases. The aerial part of *Herba Epimedii* (Yinyanghuo in Chinese, Figure 1) has long been used for “strengthening reproductive function and the skeletal system” [1,2]. In Chinese medicine, Yinyanghuo actually refers to herbs of multiple species. There are five *Epimedii* species classified under the same yinyanghuo herb name, according to the Chinese Pharmacopoeia, namely *E. brevicornu *Maxim, *E. koreanum *Nakai, *E. sagittatim *(Sieb & Zucc.) Maxim, *E. pubescens *Maxim and *E. wushanense *T.S. Ying [3]. On top of that, *E. acuminatum* Franch and *E. leptorrhizum* Stearn are also major species in *Epimedii* herb production, and at least 20 species of *Epimedii* herbs are used on the market [4]. *Herba Epimedii* was proven to be an effective remedy for cardiovascular diseases, osteoporosis and for improving sexual and neurological functions [5,6,7]. It was verified that the effectiveness of *Herba Epimedii* is highly related to its anti-oxidant ingredients. The association of free radical damage and oxidative stress with cardiovascular diseases, ageing and neurological defects is well documented (Table 1). Thus, it is essential to identify the effective anti-oxidative principles in *Herba Epimedii* in light of the prospect of developing effective drugs against these life-threatening diseases.

**Table 1:** Different Components and Activities of *Herba Epimedii.*

Component	Anti-oxidative Activity	Reference
Total Flavonoid of *Epimedium*(TFE)	1. Inhibition of croton oil-induced ear edema and granuloma.	[7]
	2. Inhibition of acetic acid-induced vascular permeability.	
	3. Inhibition of carrageenin-induced hind paw edema.	
	4. Inhibition of primary and secondary inflammation in adjuvant arthritis.	
	5. Inhibition of prostaglandin E and malondialdehyde product.	
	6. Enhancement of catalase.	
	7. Protection of H_2_O_2_-induced lesions in cardiocytes including inhibition of cardiocyte proliferation and increase of cardiocyte apoptosis.	[8,9]
Icarrin	1. Protection against free radical-induced damage of DNA.	[10]
	2. Protection against free radical-induced peroxidation of polyunsaturated fatty acids in low-density lipoprotein and cellular membrane	[11]
	3. Protection against H_2_O_2_-induced cell oxidative injury in vein endothelial cells.	[17]
	4. Protection against β-amyloid neurotoxicity	[20,21]
Polysaccharide	1. Reduction of serum and liver levels of lipoperoxide in aged mice and rats and lipofuscin in cardiac muscle of aged mice.	[23,24]
	2. Increase in activities of superoxidase dismutase and glutathione peroxidase	
Vitamin C	1. Maintainence of normal vasodilatory mechanism controlled by endothelial cells.	[25]
	2. Reduction of blood pressure.	
	3. Enhancement of iron absorption in gastrointestinal tract.	

Highly-reactive free radicals are by-products in uncoupled electron flow in respiration and they rapidly attack molecules in nearby cells and damage lipids in cell membranes, proteins in tissues and DNA. This will result in membrane damage, protein modification and DNA damage and is highly related to the ageing process and degenerative diseases such as cancer and heart diseases [8].

In human, endogenous anti-oxidative enzymes, such as superoxide dismutase, have evolved to prevent the accumulation of free-radicals by metabolizing the free-radicals formed. However, this protection is always far from being sufficient. Anti-oxidants from herbal sources can provide additional power to enhance our bodies’ ability in combating free radicals formed in the body. Flavonoids are formed in plants from aromatic amino acids like phenylalanine and tyrosine. Flavonoids work as anti-oxidants by increasing the level of enzymes responsible for metabolism of free-radicals and act as anti-oxidants by virtue of the electron-donating group of flavonoids. By these mechanisms, *Herba Epimedii* combats free-radicals and induces different mechanisms against pathological situations [8]. In this review, we will cover research progress in the pharmacology of *Herba Epimedii* and its components.

## 2. Anti-Oxidizative effect of different ingredients in *Herba Epimedii*

**Figure 2:**
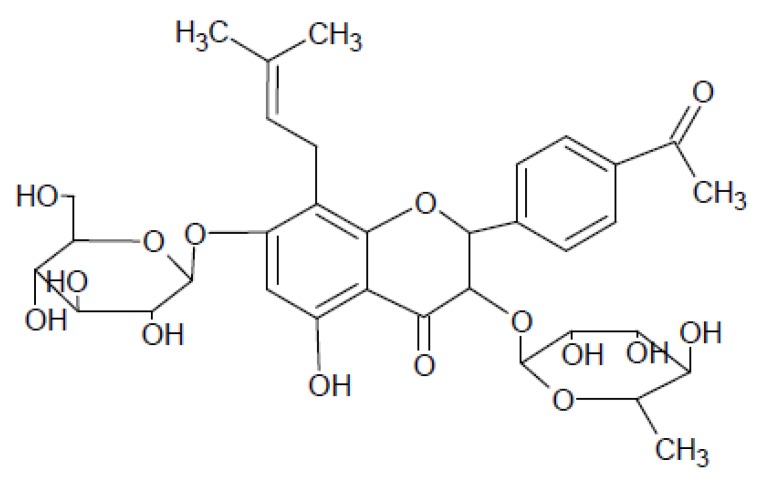
Icarrin (2-(4’-methoxyphenyl)-3-rhamnosido-5-hydroxyl-7glucosido-8-(3’-methyl-2-butylenyl)-4-chromanone).

The main constituents of *Herba Epimedii* are flavonoids, an important class of natural anti-oxidants. Therefore, the Total Flavonoid of *Epimedium* (TFE) has been well investigated for its antioxidant properties (Table 1). Among the flavonoids present in *Herba Epimedii*, icarrin (Figure 2) is recognized as the major pharmacologically active component [9] and has been further extracted for detailed studies. Besides, other components such as polysaccharides and vitamin C also contribute to the anti-oxidative ability of *Herba Epimedii* in different organs. In the following sections, the different components of *Herba Epimedii* will be discussed.

### 2.1. Total flavonoids of Epimedium

By using column chromatography, flavonoids were isolated from *Herba Epimedii* of different origins. From *E. wushanense*, diphylloside A, epimedoside A, epimedin C, icarrin, epimedoside C, icarisoside A and desmethylanhydroicaritin have been isolated [10]. The content of TFE varies according to spieces, geographical origin [11,12] or time of harvest of the herbs [12]. It is reported that more than 60 different flavoind compounds can be isolated from *Herba Epimedii* [13]. In-depth research into the flavonoid content of *Herba Epimedii* has been conducted by researchers in mainland China. Zhou *et al.* reported the TFE content in four speices of herba epimedii, namely *E. pubescens* Maxim, *E. koreanum *Nakai, *E. brevicomum* Maxim and *E. sagittatum *(Sieb. et Zucc.) Maxim which ranged from 29.72 mg/g to 82.63 mg/g (around 2.972% to 8.263%) depending on the speices and source of herbs [11]. Other research reported a TFE content ranging from 0.927% to 15.2% in nine speices of epimedium herbs depending, as mentioned, on the particular species, harvesting time and source of origin [12].

#### 2.1.1. Anti-oxidative effect in anti-inflammatory effects of total flavonoid of *Epimedium* (TFE)

TFE is observed to inhibit the chronic and acute inflammatory effects in mice. Inflammatory pathologies such as ear edema and granuloma induced by croton oil, increased vascular permeability induced by acetic acid and hind paw edema induced by carrageenin are all significantly inhibited by TFE. TFE also exerts a suppressive action on primary and secondary inflammation in adjuvant arthritis. This anti-inflammatory ability of TFE is believed to be related to its anti-oxidant ability. TFE significantly inhibits prostaglandin E and the metabolic product of lipid peroxidation, malondialdehyde (MDA) and also enhances the activity of free-radical scavenging enzyme, catalase (CAT), in murine erythrocytes [14].

#### 2.1.2. Anti-oxidative effect of total flavonoids of* Epimedium*(TFE) in cardiocytes

TFE exerts a protective effect against cardiocyte lesions induced by H_2_O_2_. The cardiocytes are isolated from neonatal rats and subsequently culture *in vitro*. The proliferative status of cardiocytes under the influence of H_2_O_2 _and TFE is studied by MTT proliferation assay. Also, apoptosis of cardiocytes is studied by flow cytometry and the morphological changes are observed under a transmission electron microscope. Treatment with H_2_O_2_ leads to inhibition of cardiocyte proliferation in a dose-dependent way and changes the cardiomyocyte ultrastructure such as nuclear fragmentation. The apoptotic rate is (23.94 ± 3.52)%, which is higher than that of control cells (1.98 ± 1.22%) at twelve hours after administration of H_2_O_2_. Treatment of TFE is able to reverse the apoptotic effect of cardiocytes at the doses of 100, 200 and 400 mg/L respectively [(15.12 ± 3.01)% , (9.38 ± 3.14) % and (4.49 ± 0.52)% cells undergoing apoptosis] [15].

Another animal experiment also illustrates the protective effect of TFE on the heart in acute myocardial anoxia. The mean survival time and capacity of cardiac oxygen consumption under ordinary pressure in mice are recorded and time of disappearance of electrocardiographic sign is measured after clamping the trachea. TFE administration prior to the experiment is capable of prolonging the mean survival time and decreasing the time of disappearance of electrocardiographic sign [16].

### 2.2. Icarrin

Icarrin (ICA) is a flavonoid with structural formula C_33_H_40_O_15_ and molecular weight of 676.67. It is suggested that the icarrin is the major bioactive components and exists in many speices of *Epimedii *plants. The discovery of icarrin from the species *E. macranthum* Morr et Decne by Japanese researchers can be traced back to 1935 [17]. Its content in *Herba Epimedii *has been reported by different groups. Like TFE, the content of icarrin varies among species and sources. The icarrin content ranged from 0 to 2.732% among nine species, in which the *E. pubescens* Maxim showed the highest content, while *E. leptorrhizum *Stearn showed none [12]. Another group reports the flavonoid content from 17 species, in which icarrin content ranged from 0–14.24 mg/g [18]. This suggests certain kinds of *Epimedii* plants may serve as better herbal materials for pharmaceutics purposes. 

#### 2.2.1. Anti-oxidative effect of icarrin on radical-induced oxidative damage of DNA

Icarrin protects DNA against radical-induced oxidative damage in a concentration-dependent manner. In the experiment, the oxidative damage of DNA is measured by the formation of carbonyl compounds that can react with thiobarbituric acid (TBA) to form thiobarbituric acid reactive substance (TBARS) [19].

#### 2.2.2. Anti-oxidative effect of icarrin against free-radical-induced peroxidation of polyunsaturated fatty acids in low-density lipoprotein and cellular membrane

Icarrin protects polyunsaturated fatty acids in erythrocyte cellular membrane and prevents erythrocytes from undergoing haemolysis. Erythrocyte membranes contain abundant polysaturated fatty acids that are prone to peroxidation by free-radicals generated from the decomposition of a water-soluble azo compound, 2,2’-azobis(2-amidinopropane hydrochloride) (AAPH). The degree of peroxidation is measured by the haemoglobin concentration outside the erythrocytes. It is found that icarrin dose-dependently protects erythrocytes against free-radical peroxidation, which is attributed to the presence of hydroxyl group at the 5-position when the anti-oxidative activities of structural analogs are compared [20].

#### 2.2.3. Anti-oxidative effects of icarrin on vein endothelial cell oxidative injury induced by H_2_O_2_

Icarrin is able to reduce the extent of oxidative injury in endothelial cells. Oxidative injury induced by ischemia/ hypoxia, reperfusion and inflammation can lead to cardiac and endothelial cell apoptosis [21,22,23,24]. Such apoptosis of endothelial cells would disrupt the integrity of the endothelium monolayer that is crucial to prevent vascular leakage and formation of atherosclerosis [25]. In the experiment, eighteen-hour treatment with 750 μmol L^-1^ H_2_O_2_ significantly reduces the viability of human umbilical vein ECV-304 endothelial cells and induces apoptotic features such as distinct morphological alteration and increases caspase-3 expression. Pretreatment with icarrin reverses the injury and apoptosis in a dose-dependent manner and the expression of caspase-3 is also reduced [26].

#### 2.2.4. Anti-oxidative effects against neurotoxicity of β-amyloid

Icarrin is found to inhibit the neurotoxicity of β-amyloid peptide, which is a neurotoxic species associated with the pathogenesis of Alzheimer’s disease (AD). It enters the mitochondria to induce the generation of reactive oxygen species and subsequent free-radical attacks on protein, DNA, RNA and lipid. This eventually leads to neuronal death [27,28]. The ability of spatial learning and memory of the animals is tested by using Morris water maze. Icarrin significantly decreases the mean escape latency and searching distance in place navigation test while it also increases the searching time and searching distance in the quadrant in comparison to mice unilaterally injected with amyloid β-protein. Through immunohistochemistry and real time RT-PCR analysis, it discloses that icarrin significantly decreases the content of β-amyloid and mRNA levels of β-secretase in the hippocampus, which is a key enzyme in generation of β-amyloid peptide from amyloid precursor protein. Also, mRNA level of an antioxidant enzyme, superoxide dismutase-2, is increased in the presence of icarrin [29].

Another experiment reveals the protective ability of icarrin against neurotoxicity of β-amyloid by up-regulating cocaine-regulated and amphetamine-regulated transcripts. Also, this up-regulation is dependent on the mitogen-activated protein kinase/extracellular signal-regulated kinase (MAPK/ERK) pathway. In the experiment, mRNA and protein levels of cocaine and amphetamine-regulated transcript (CART) are increased in neuronal culture pretreated with β-amyloid peptide. This induction and protective effect are suppressed by extracellular signal-regulated kinase inhibitor and CART-RNA interference. This further experiment explores the mechanism and pathway involved in the neuroprotective action of icarrin [30].

Icarrin protects against cognitive deficits induced by chronic cerebral hypofusion in rats through its antioxidant effects, and effects on the circulatory and cholinergic systems [31]. It inhibits release of reactive oxygen species, nitric oxide, prostaglandin E and mRNA expression of proinflammatory cytokines in microglia [32].

### 2.3. Polysaccharides

Polysaccharides in *Herba Epimedii* are composed of monosaccharides including mannose, glucose, 6-deoxymannose, galactose, arabinose and galacturonic acid. The polysaccharide content varies among *Herba Epimedii* of different origins, ranging from 18.64% to 31.11% [33].

#### 2.3.1. Anti-oxidative effect of polysaccharide

Polysaccharides increase the anti-oxidant ability of erythrocytes by increasing the activities of intracellular key enzymes responsible for scavenging free-radicals [34]. In one experiment, male Sprague-Dawley rats are employed to investigate the effect of polysaccharide for consecutive 50-day treatment. Polysaccharides help reduce the serum and liver content of lipoperoxide (LPO) in aged mice and rats, together with the content of lipofuscin (LF) in cardiac muscle of aged mice [34]. These two parameters reflect the lipid oxidation level. The higher level of LF in cardiac muscle, the higher is the level of oxidation of lipid.

Superoxidase dismutase (SOD) and glutathione peroxidase (GSH-Px) activity levels are also increased after administration of the polysaccharide [34]. The two enzymes are important in scavenging free-radicals inside the cells. It is also found that SOD activity is induced in chickens in a dose-dependent manner [35].

### 2.4. Vitamin C

Vitamin C has long been known as an effective natural antioxidant given its electron-donating property. The anti-oxidant properties of vitamin C have been established in *in vitro* experiments [36]. Vitamin C is a significant component in *Herba Epimedii* and contributes to the anti-oxidant properties of *Herba Epimedii*. Vitamin C in *Herba Epimedii* is determined by ultraviolet spectrophotometry at 243 nm. 1.0% HCl solution is used for extraction of vitamin C from *Epimedium* leaves. A variation of the contents of vitamin C is found among the cultivated samples of *Herba Epimedii *from different sources (*E. sagittatum, E. myrianthum and E. doluchostemon*) [37]. The importance of vitamin C has only been demonstrated in prevention of life-threatening scurvy disease in clinical studies. However, pre-clinical investigations employing *in vitro* experiments suggest the importance of vitamin C in maintaining normal vasodilatory mechanism controlled by endothelial cells, low blood pressure and enhancement of iron absorption in gastrointestinal tract [36]. The role of vitamin C as an anti-oxidant in *Herba Epimedii *is yet to be explored in the future.

In addition to the aforementioned activities, flavonoids from *E. brevicomum* prevents bone loss in postmenopausal women [38]. An aqueous extract of *E. saggittatum* prevents ovariectomy-induced bone loss in rats [39]. Total flavonoids of *Herba Epimedii* increase the expression of core binding factor alpha 1 which regulates the differentiation of osteoblastic precursors and activity of mature osteoblasts [40]. A water extract of *Herba Epimedii* lowers serum levels of total cholesterol and triglycerides, but elevates serum estradiol levels in postmenopausal women [41]. Well processed *Herba Epimedii *improves sexuality in male rats [42]. Icariside II from *E. koreanum* roots has antioxidant activity and inhibits melanogenesis and induces apoptosis in human PC-3 prostate cancer cells [43]. 

## 3. Conclusions

Numerous studies have established the anti-oxidative ability of *Herba Epimedii* against different oxdative stress conditions *in vitro* and *in vivo*. The ability of flavonoids to increase antioxidation enzymes and inhibit lipid peroxdiation product has be verified in murine erythrocytes. TFE is also able to reduce the cardiocytes lesion induced by H_2_O_2 _and exerts protective effect on the heart in acute myocardial anoxia. Icarrin, as a major bioactive component, is also demonstrated to protect against radical induced damage to DNA and peroxidation of polyunsaturated fatty acids. Protective effects of icarrin on the H_2_O_2_ induced stress and neuroprotection against β-amyloid was also reported. The stimulation of enzymes responsible for scavenging of free radicals and antioxidation by polysaccharides from *Epimedii* also add value to the pharmaceutical potential of the herb. Further clinical applications of this traditional herbal medicine may be developed on the basis of these pharmacological mechanisms found in *in vivo* and *in vitro*. Diseases related to oxidative-stress may also serve as target for further research.
